# Pattern of Amino Acid Substitutions in Transmembrane Domains of *β*-Barrel Membrane Proteins for Detecting Remote Homologs in Bacteria and Mitochondria

**DOI:** 10.1371/journal.pone.0026400

**Published:** 2011-11-01

**Authors:** David Jimenez-Morales, Jie Liang

**Affiliations:** Department of Bioengineering, University of Illinois at Chicago, Chicago, Illinois, United States of America; University of Georgia, United States of America

## Abstract

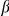
-barrel membrane proteins play an important role in controlling the exchange and transport of ions and organic molecules across bacterial and mitochondrial outer membranes. They are also major regulators of apoptosis and are important determinants of bacterial virulence. In contrast to 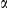-helical membrane proteins, their evolutionary pattern of residue substitutions has not been quantified, and there are no scoring matrices appropriate for their detection through sequence alignment. Using a Bayesian Monte Carlo estimator, we have calculated the instantaneous substitution rates of transmembrane domains of bacterial 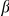-barrel membrane proteins. The scoring matrices constructed from the estimated rates, called bbTM for 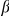-barrel Transmembrane Matrices, improve significantly the sensitivity in detecting homologs of 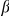-barrel membrane proteins, while avoiding erroneous selection of both soluble proteins and other membrane proteins of similar composition. The estimated evolutionary patterns are general and can detect 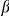-barrel membrane proteins very remote from those used for substitution rate estimation. Furthermore, despite the separation of 2–3 billion years since the proto-mitochondrion entered the proto-eukaryotic cell, mitochondria outer membrane proteins in eukaryotes can also be detected accurately using these scoring matrices derived from bacteria. This is consistent with the suggestion that there is no eukaryote-specific signals for translocation. With these matrices, remote homologs of 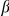-barrel membrane proteins with known structures can be reliably detected at genome scale, allowing construction of high quality structural models of their transmembrane domains, at the rate of 131 structures per template protein. The scoring matrices will be useful for identification, classification, and functional inference of membrane proteins from genome and metagenome sequencing projects. The estimated substitution pattern will also help to identify key elements important for the structural and functional integrity of 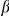-barrel membrane proteins, and will aid in the design of mutagenesis studies.

## Introduction

As one of the two classes of integral membrane proteins, 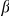-barrel membrane proteins are found in the outer membranes of gram negative bacteria, mitochondria, and chloroplasts. Because they are located in the first barrier of bacteria and are in contact with the extracellular environment, they are often key factors providing control of the diffusion, exchange, and transport of ions and organic molecules [1]–[5]. They are also involved in the transmission of signals in response to stimuli and, as enzymes, in the maintaining of the stability of the outer membrane [2], [6]. In eukaryotes, mitochondrial outer membrane proteins are part of the mitochondrial permeability transition pore (mtPTP), a major regulator of apoptosis, with important implications in cancer, degenerative diseases, and aging [7]. For example, the voltage-dependent anion channel (VDAC) is considered a promising target for anticancer treatments [8].


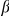
-barrel membrane proteins are also important determinants of bacterial virulence and are promising drug targets [9]–[11]. As bacterial porins enable the diffusion of hydrophilic antibiotics through outer membranes, mutation of their barrel interior is the basis of a common mechanism of bacterial drug resistance [12], [13]. 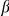-barrel membrane proteins therefore are excellent targets for developing new antibacterial drugs. A promising example is the recent discovery of a new peptidomimetic antibiotic that perturbs the critical LPS transport function of the 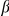-barrel membrane protein LptD [11].

The architecture and amino acid make-up of 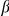-barrel membrane proteins have been well studied [2], [14]–[16]. Several methods have been developed for the detection of 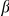-barrel membrane proteins from sequences [17]–[20]. Sequence motifs and antimotifs in transmembrane regions of 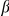-strands have also been identified, with tyrosine found to play important roles [21]. In addition, propensities of residues for different spatial regions and for inter-strand pairwise contact have been quantified [21]–[23]. A physical model of energetics based on the estimated propensities of spatial interactions enabled the identification of weakly stable regions in the TM domain, the discovery of general mechanisms of their stabilization, the prediction of oligomerization states, and the delineation protein-protein interaction interfaces [24].

A remaining challenging task is the detection and quantification of evolutionary patterns of residues embedded in the TM region. The amino acid sequences of 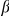-barrel membrane proteins determine how these proteins fold, insert into the membrane, and carry out their biological functions. As evolution proceeds, the set of allowed amino acid substitutions at different positions of the transmembrane segments are constrained by these requirements, which manifest as patterns of substitutions that correlates with the amino acid type, solvent accessibility, secondary structure, depth of lipid buriedness, and side-chain hydrogen bonding states [25], [26]. Currently, it is not clear how residues substitute in the outer membrane region of gram negative bacteria. In addition, whether membrane proteins in mitochondria and bacterial outer membrane show the same evolutionary pattern is unknown. Understanding of the evolutionary patterns of 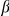-barrel membrane proteins can help us to identify key features important for their structural and functional integrity. Furthermore, it can aid in the design of mutagenesis studies [13].

Characterizing amino acid substitutions can also be used to develop scoring matrices specific for 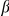-barrel membrane proteins for sequence alignment, structure prediction, and large scale database searches of remote homologs. Conventional scoring matrices used for database searches are not designed for 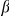-barrel membrane proteins. For example, the Pam [27] and Blosum matrices [28] were derived from large collections of multiple sequence alignments of globular proteins, and are inappropriate for studying membrane proteins [29]. A number of scoring matrices have been developed for membrane proteins. The Phat matrices are based on blocks of multi-aligned sequences of transmembrane segments and hydrophobic segments [30]. The Slim matrices are based on models of different background compositions of amino acid residues [31]. However, they are all derived for studying 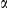-helical membrane proteins.

To capture the pattern of amino acid substitutions of 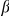-barrel membrane proteins, we have estimated substitution rates of amino acids in the transmembrane segments. Our approach was based on a Bayesian Monte Carlo method [32]. We selected a representative set of eleven proteins with known structures and with pairwise sequence identities below 20%. For each protein, substitution rates were estimated for residues in the transmembrane segments. These estimated rates show characteristic patterns that are unique to 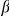-barrel membrane proteins. From these estimated rates, we derived scoring matrices useful for sequence alignment and for detecting remote homologs of 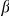-barrel membrane proteins. Results of database searches showed that these scoring matrices can significantly improve reliability in detection of 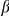-barrel membrane proteins by eliminating errors of selecting soluble proteins as well as other membrane proteins of similar composition.

This paper is organized as follows. We first describe the pattern of amino acid substitutions found for TM segments of bacterial 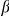-barrel membrane proteins. We then discuss how scoring matrices derived from the estimated substitution rates can be used for reliable detection of homologs. This is followed by a description of how mitochondria outer membrane proteins can also be detected using these scoring matrices derived from bacterial proteins. We then consider the implications in predicting structures of bacterial and mitochondrial membrane proteins using known structures as templates.

## Results

We use a set of 11 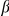-barrel membrane proteins with known structures sharing less than 20% pairwise sequence identity (Table 1). We followed the procedure of Jackups *et al* [23] and select the fragments embedded within the outer membrane region. Altogether we have 170 TM-strands. From these, we further derive two additional data sets, one for residues facing the interior of the barrel, and another for residues facing the lipid environment of the outer membrane. These three data sets are termed TM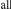, TM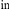, and TM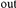, respectively. More details about these 11 proteins, their homologous proteins and phylogenetic trees can be found in Figure S1.

**Table 1:** The 11 template proteins, their composition, and hydrophobicity index values.

	# of Residues and TM Strands			Hydrophobicity Index (GES)		
PDB	TM 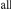 /Total/# Strands	TM 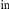	TM 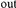	TM 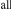	TM 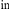	TM 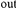
1A0S	172/413/18	84	87	−0.54	−1.66	0.52
1BXW	84/172/8	42	42	−0.05	−1.76	1.66
1E54	139/332/16	70	69	−0.33	−1.8	1.17
1FEP	206/724/22	102	104	−0.67	−2.25	0.87
1I78	102/297/10	50	51	−0.11	−1.99	1.71
1KMO	217/774/22	108	109	−0.94	−2.6	0.7
1NQE	220/549/22	111	109	−0.87	−2.47	0.77
1QD6	124/240/12	59	64	−0.63	−2.64	1.16
1QJ8	75/148/8	35	40	0.2	−1.02	1.27
2MPR	178/427/16	90	87	−0.75	−2.5	1.04
2OMF	153/340/16	76	77	−0.66	−2.38	1.04
Mean	152/401/16	75	76	−0.49	−2.10	1.08

TM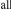: number of residues in the TM region; Total: total number of residue in the protein; # Strands: number of 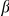-strands in the TM region; TM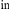: number of residues in the TM in-facing region; and TM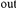: number of residues in the TM lipid out-facing region. The hydrophobicity is measured by the GES index [33], with negative values representing polarity and positive values hydrophobicity.

The transmembrane segments as a whole have moderate polarity (−0.49 by the GES scale [33]). However, the in-facing residues in TM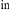 are strongly polar (polarity of −2.10) and the out-facing residues in TM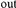 are strongly hydrophobic (+1.08) (Table 1).

### Pattern of amino acid substitutions in transmembrane segments

#### Overall pattern

**Figure 1:**
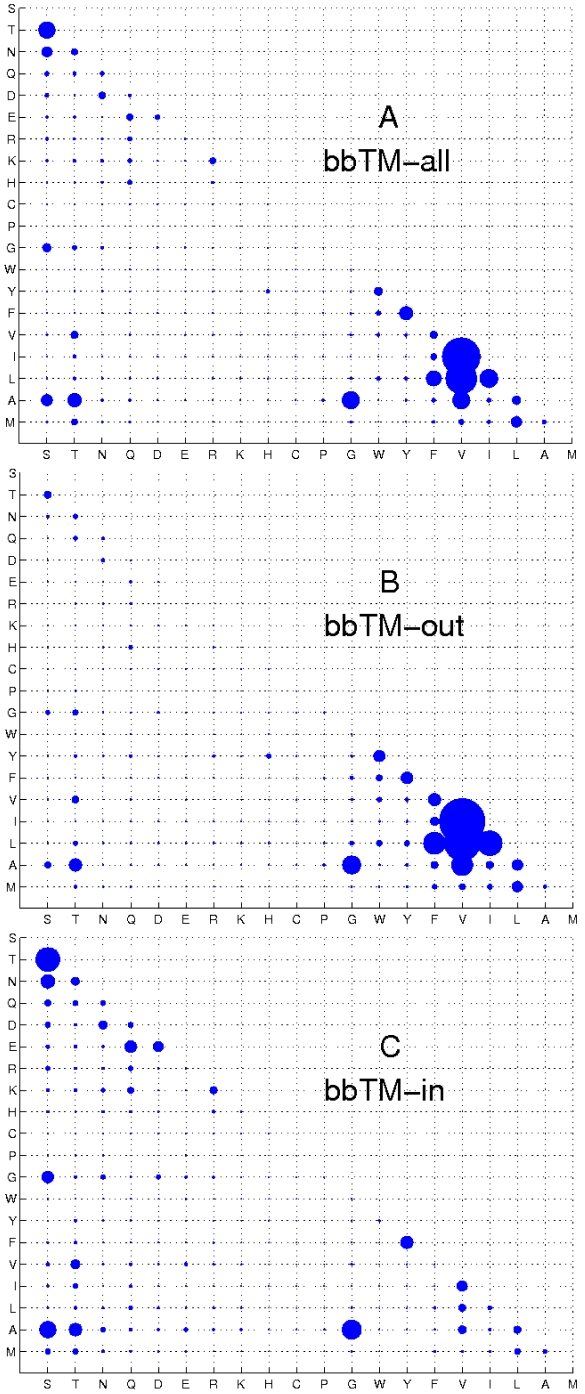
Estimated amino acid substitution rates. Estimated instantaneous rates of substitution for residues in the TM segments and at different TM interfaces from 11 template 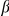-barrel membrane proteins. The size of the bubble is proportional to the value of the estimated substitution rate. The instantaneous substitution rates (A) for all TM residues (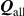); (B) for residues out-facing the membrane (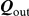); and (C) for residues in-facing the membrane (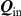).

The general pattern of amino acid residue substitutions observed for residues in the TM region is shown in Figure 1A (see also Figure S2). Residues with similar physiochemical properties often exchange with each other. V has overall the highest degree of substitutions, and exchanges mostly with L, I, and A. The instantaneous rate of V-I substitution is 194 in the unit of 10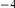 expected residue changes per 100 site between sequences. The value for V-L is 131. L and I have the next highest overall degree of substitutions. In addition to V, they frequently exchange between themselves (I-L: 44), and substitute with other hydrophobic residues (L-M:16, L-A:10, I-A:3), and the aromatic residue F (L-F:32, I-F:5).

Small polar residues S and T substitute mostly between themselves (S-T: 38), with the small residues A (25 for T-A, 18 for S-A) and G (S-G:9, T-G:3). Exchanges also occur with N (T-N:6, S-N:15).

Among large polar residues, Q shows overall lower substitutions, but with a broader number of residue types, *e.g.*, with charged residues E (E-Q:6), H (H-Q:53), R (R-Q:3) and K (K-Q:3), and with polar residues S (S-Q: 3) and N (N-Q: 3). Residue N readily substitutes with polar residues S (S-N:15), T (T-N:6) and Q (Q-N:3), and with the charged residue D (D-N:7).

Aromatic residues most likely substitute among themselves (*e.g.*, Y-F:25, Y-W:9, W-F:3). Residue F has the broadest range of substitutions among aromatics and exchanges with L (F-L:32), V (F-V:8), I (F-I:5), W (F-W:3) and A (F-A:3).

The most abundant residue in the transmembrane segments of 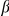-barrel membrane proteins is residue G. This residue overall experiences little substitutions. The relatively few substitutions are with A (A-G:40), S (S-G:9) and T(T-G:3).

#### Substitution rate of residues facing the outer membrane lipids

The pattern of substitutions for residues facing the outer membrane lipids (TM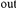) is shown in Figure 1B (Figure S2). The most common substitutions observed are between hydrophobic residues, namely, substitutions among V, I, L, A and F. For example, V has the highest degree in overall substitutions, showing large values with V-I (275), V-L (168), V-A (61) and V-F (22).

L is the most abundant residue in the TM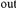 region. It predominantly substitutes with non-polar residues, *i.e.*, L-V (168), L-I (82), L-A (16) and L-M (16). Other observed exchanges are with aromatic residues (*e.g.*, L-F:65, L-W:4, L-Y:4) and T (L-T:3). Residue I also exchanges mostly with other non-polar amino acids (I-V:275, I-L:82, I-F:10, I-A:8 and I-M:4).

Residue A has a broad range of substitutions at the TM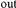 interface. It mostly exchanges with other hydrophobic or small amino acids, including V (V-A:61), G (G-A:46), L (L-A:16), F(F-A:7), and I(I-A:5). Notable exceptions are with polar residues T (T-A:22) and S (S-A:6).

Among aromatic residues, Y is well conserved at the lipid-facing surface of 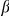-barrel membrane proteins. This is reflected by the relatively small number of its substitutions. It is the second most frequent amino acid residue at the lipid interface, and contributes significantly to the formation of the aromatic girdle [34], a prominent feature of 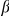-barrel membrane proteins. Substitutions of Y with other aromatic residues are most common (Y-F:20, Y-W:18), and to a small degree also with L (Y-L:4) and H (Y-H:3). Aromatic residue F is less abundant compared to Y, but experiences more varieties of substitutions, mostly with hydrophobic residues: F-L (65), F-V (22), F-Y (20), F-I (10), F-A (7), F-W (5) and F-M (4). The aromatic residue W resides mostly in the TM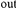 region. Its pattern of substitution is very restricted and mostly substitutes with Y (W-Y:14).

The predominant polar residue at the TM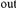 interface is T. It substitutes with non-polar amino acids (T-A:17, T-V:6, T-G:4) and the polar residue S (7). In contrast, polar residue S substitutes only with T (7) and A (5). Among ionizable residues, E has low abundance in the TM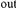 interface and low tendency for substitutions.

Finally, the only substitution observed for G in this interface are mainly with A (46), and to a less extent with T (4) and S (3).

#### Substitution rates of residues facing the interior of the barrel

The pattern of substitutions for residues facing the interior of the barrel (TM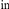) differs significantly from that of the TM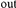 region (Figure 1C, Figure S2). Small residues S, T, and A experienced most frequent substitutions (S-T:77, S-A:38, and S-N:26). Q and N have a much higher presence at the TM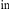 region, with increased substitutions.

Ionizable residues such as E, R, K and D are more abundant in the TM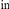 interface. Most of them do not substitute with other residues. For example, E is among the most abundant residues in the TM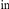 region. It is well conserved, substituting mostly only with Q (E-Q:20) and the other negatively charged residue D (E-D:14). Similar patterns are found for the residues R and K, which exchange mostly between themselves (R-K:8) and with polar residue Q (K-Q:5, R-Q:4). The lack of substitutions of ionizable residues suggests that they play a significant role in the function of the 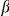-barrel membrane proteins and are under strong purifying selection pressure.

The pattern of substitutions for hydrophobic residues is somewhat different at this interface. Although V, I, L and M mostly exchange amongst themselves, they also exchanges more frequently with polar residues such as T, in contrast to what is found in the TM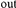 region.

The most abundant residue at this interface is G. Its substitution pattern shows some similarities with G at the lipid interface, although a larger number of substitutions is observed with S (S-G:19).

### Residues similar in substitution pattern

**Figure 2:**
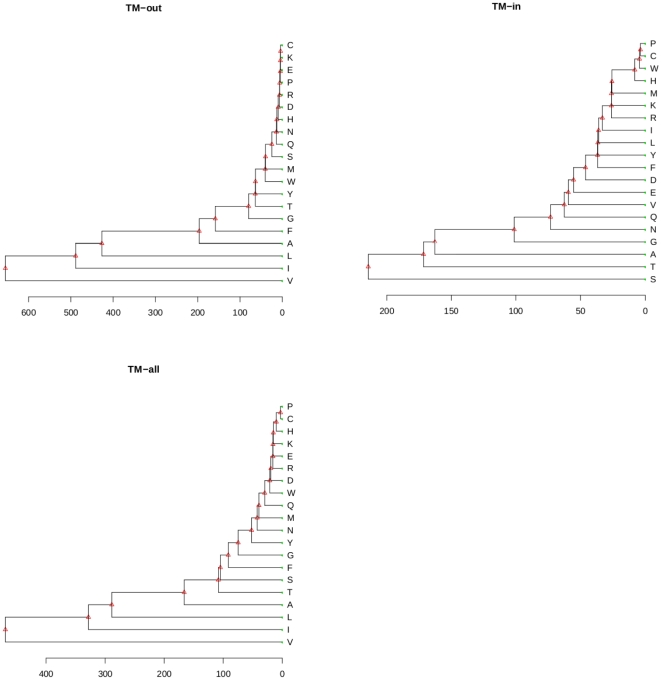
Similarity in substitution pattern for residues in the TM region of 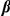
 **-barrel membrane proteins.** Clustering trees showing grouping of residues in the transmembrane regions by similarity in substitution patterns. Residues are clustered by pairwise euclidean distance between the 19-dimensional vectors of instantaneous rates of residue substitutions.

To identify residues that behave similarly in their patterns of substitutions, we carried out clustering analysis based on the substitution profile of the 20 amino acids. For each amino acid residue, we collected the substitution rates of replacing this residue type with each of the other 19 residue types. These rates form a 19-dimensional vector. As each of the twenty amino acid types has its own vector, we collected a set of twenty vectors and calculated the Euclidean distances between all pairs of vectors. We then carried out single-linkage hierarchical clustering analysis. This is repeated for each interface region and for the entire TM region. The resulting clustering trees are shown in Figure 2.

There is clear grouping of residues in the clustering tree for the TM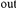 region, which correlates well with the physical-chemical properties of residues. A tight cluster consisting of ionizable and polar residues (*i.e.*, K, E, R, Q, D, N, H, S), along with infrequently observed residues (C and P) arise naturally. The aromatic residues W and Y are grouped together, and the small residues G and T are also grouped together. The branched hydrophobic residues (I, V, and L) are also found to cluster together, and are all very different from other residues in their behavior of substitution. Aromatic residue F seems to behave differently from Y and W in substitution, and is grouped closer to the hydrophobic amino acids (A, I, L, V) enriched in the lipid-facing interface. Distances are much larger in this interface due to the larger values of substitutions for hydrophobic residues.

The general pattern for the TM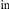 region is different. Residues have overall lower degree of substitutions in this interface, showing closer distances. Hydrophobic residues (L, I, V), which substitute very differently from other residues in the TM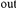 region, are now clustered much closer to other residues. The small residues S, T, A and G, along with N and Q, are grouped together, and show significantly different substitution pattern from other residues.

The hierarchical tree for the TM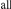 interface shows stronger similarities to the tree for the TM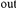 region, reflecting the fact that the substitution pattern in the TM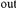 region dominates. Overall, the hydrophobic residues are found to cluster together (V, I, L, and A). The tight cluster of polar residues and infrequently observed residues are similar to those which are observed in the TM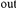 region.

### Detection of homologs of 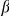-barrel membrane proteins

The estimated amino acid substitution rates can be used to construct scoring matrices for sequence alignment and for large scale database search of homologs of 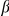-barrel membrane proteins. When scoring matrices accurately reflect the evolutionary history of the underlying protein sequences, the detection of homologs usually can be improved significantly [27].

Three sets of scoring matrices were derived from the estimated substitution rates (see Figure S3): scoring matrices for the whole TM segments (bbTM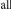, for 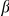-barrel Transmembrane Matrices), for residues facing the interior of the barrel (bbTM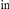); and for residues facing the lipid outer membrane (bbTM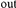) (See Figure S3 for details). These scoring matrices were assessed for performance through Blast searches against several databases using the TM fragments of a set of 20 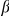-barrel membrane proteins with known structures as templates (see Dataset S1).

To obtain objective evaluation, we constructed a “true-positive” data set containing known and predicted 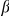-barrel membrane proteins, as well as a data set of negative controls consisting of randomized sequences of 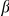-barrel and 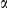-helical membrane proteins. We created the first data set by combining 2,130 predicted 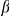-barrel membrane proteins sequences from the PROFtmb database constructed by Bigelow et al [35], with an additional 1,266 sequences annotated as bacterial outer membrane proteins in the Uniprot database [36]. We excluded those proteins with more than 90% identity with the 11 proteins from which we estimated the substitution rates. After removal of redundant sequences, we have a total of 3,079 sequences (see Dataset S2). The second data set consists of random sequences obtained from fully shuffled sequences of 385 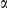-helical and 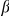-barrel membrane proteins from different organisms. These random sequences preserve the same amino acid composition as membrane proteins. Assuming none of the randomized sequences resemble a true 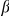-barrel membrane protein, they form a challenging set of “true negatives” (see Dataset S2).

Two additional data set were constructed from the Uniprot database [36]. The first data set consists of membrane proteins with a different architecture (non-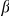-barrel). These were selected based on annotations of “SUBCELLULAR LOCATION: Cell membrane” from *Eukaryota* and *Archaea*. We make the reasonable assumption that 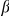-barrel membrane proteins cannot be found in the cellular membrane of these organisms, and all these membrane proteins are expected to adopt a different three dimensional topology. In total, 10,951 these other-membrane protein sequences were included in the data set (called oMBp, for other MemBrane proteins, Dataset S3). The second data set consists of globular protein sequences. We selected from Uniprot proteins with annotations that lack the word “membrane”. In total, 127,485 globular protein sequences were included in the data set (called Globular, Dataset S4).

We use the concatenated transmembrane segments of 11 proteins from which the scoring matrices were derived, along with an additional 9 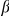-barrel membrane proteins, as templates to search the databases for homologs. These 20 proteins share less than 20% pairwise sequence identity. Our goal was to detect homologs of 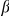-barrel membrane proteins with accuracy and specificity. We use the simple criterion that resulting hits from Blast searches using these customized scoring matrices must have 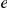-values smaller than 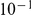. 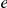-value measures the statistical significance of matched sequences from database search. It gives the expected total number of hits in a database search one would find by random chance [37]. We therefore set the threshold of 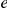-value to be 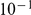. We also require that the alignment must be of a minimum length. Since 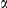-hemolysin has the smallest number of strands in forming a 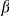-barrel membrane, we require that the matched sequence must be at least of the length of about two transmembrane segments. In 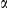-hemolysin, two TM strands form a hair-pin, and seven repeats of hair-pins form the 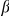-barrel membrane protein [38]. Assuming that at least 5 amino acids need to be matched in a TM strand, an hairpin would require 10 amino acids to be matched. We therefore require that an alignment should be no less than 10 residues.

#### Evaluation of specificity using random sequences, other (non-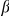-barrel) membrane proteins and globular (non-membrane) protein sequences

We first carry out a test of specificity. Results of Blast searches against the randomized database are shown in Table 2. A perfectly discriminative scoring matrix should not select any sequence from the database of randomized membrane proteins. Search results using the bbTM matrices showed excellent specificity, with no sequences retrieved from the random database. Although the default matrix Blosum62 used in Blast searches were designed for soluble proteins and is not suitable for homology detection of membrane proteins, it did not retrieved sequences from the random database.

**Table 2:** Specificity of scoring matrices in detecting 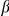-barrel membrane proteins.

*e*-value	bbTM_all_	bbTM_in_	bbTM_out_	Blosum62	Phat7573	Slim161	Pam250
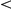 10 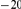	0	0	0	0	0	0	0
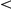 10 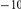	0	0	0	0	0	0	0
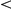 10 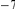	0	0	0	0	0	0	0
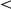 10 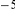	0	0	0	0	0	20	0
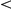 10 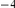	0	0	0	0	0	52	0
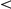 10 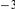	0	0	0	0	0	142	5
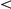 10 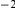	0	0	0	0	5	319	42
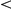 10 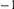	0	0	0	0	45	689	181

Cumulative number of random sequences incorrectly identified as homologs of 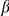-barrel membrane proteins at different 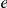-value resulting from Blast searches against a database of 362 randomized membrane proteins sequences using as queries the concatenated transmembrane segments of 20 template 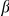-barrel membrane proteins.

The scoring matrix Phat constructed for helical membrane proteins does not work well for 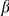-barrel membrane proteins. It selected a total of 45 random sequences with 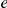-values less than 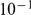, five of which with 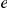-value in the range of 10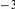 to 10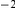. That is, 12.4% of the random sequences were mistakenly identified as membrane proteins. The performance of another scoring matrix, Slim, constructed for helical membrane proteins, also had poor performance: random sequences started to be selected at the significant 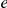-values in the range of 10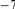 to 10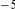, with a total of 689 random sequences selected at the 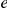-values less than 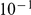, using 20 proteins as query sequences. Similarly, Blast searches using the classical Pam250 matrix resulted in 181 random sequences with significant 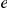-values less than 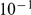.

When searches were carried out against the data set of other membrane proteins (oMBp) and the data set of globular proteins (Globular), the matrices bbTM and Blosum62 showed excellent specificity, with no sequences erroneously identified as 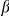-barrel membrane proteins (Table 3). In contrast, varying numbers of other membrane proteins and soluble proteins were erroneously identified as 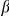-barrel membrane proteins when Pam250, Phat and Slim matrices were used. Among these, the Slim matrix resulted in a very large number (1,780) of misidentified non-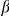-membrane proteins.

**Table 3:** Specificity of scoring matrices: Blast searches against a data set of membrane proteins with other architecture and a data set of globular proteins (oMBp/Globular).

*e*-value	bbTM_all_	bbTM_in_	bbTM_out_	Blosum62	Phat7573	Slim161	Pam250
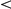 10 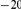	0/0	0/0	0/0	0/0	0/0	0/0	0/0
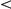 10 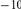	0/0	0/0	0/0	0/0	0/0	0/0	0/0
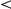 10 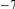	0/0	0/0	0/0	0/0	0/0	0/0	0/0
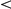 10 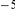	0/0	0/0	0/0	0/0	0/0	2/2	0/0
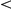 10 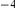	0/0	0/0	0/0	0/0	0/0	21/3	0/0
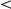 10 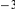	0/0	0/0	0/0	0/0	0/1	98/5	1/1
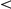 10 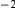	0/0	0/0	0/0	0/0	3/6	457/13	3/2
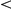 10 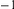	0/0	0/0	0/0	0/0	13/26	1780/42	28/31

Cumulative number of sequences of membrane proteins with other architecture and globular protein sequences incorrectly identified as homologs of 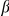-barrel membrane proteins at different 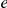-values resulting from Blast searches against the oMBp/Globular data set. The number of sequences part of oMBp is 10,951 (1,061 from *Archaea* and 9,890 from *Eukaryota*). The size of the data set Globular is 127,485 globular protein sequences (16,814 *Archaea* and 110,671 *Eukaryota*). We used as queries the concatenated transmembrane sequences of the 20 template proteins.

We conclude that the scoring matrices Phat, Slim, and Pam are not suitable for database search of 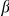-barrel membrane proteins.

#### Detection of outer membrane proteins

Next we performed Blast searches against the “true-positive” database of outer membrane proteins. Search results are shown in Table 4. bbTM matrices retrieved larger numbers of true positives, while maintaining excellent specificity as discussed before. The numbers of true positives retrieved using bbTM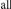, bbTM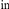, and bbTM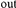 are 191, 166, and 245, respectively. Each of these proteins is related to one of the 20 query proteins and shares the same structure. As discussed earlier, the Phat and Slim scoring matrices designed for helical membrane proteins are inappropriate for search of 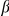-barrel membrane proteins, as they lack specificity and will select many false positives. The Blosum62 matrix performs poorly in detecting 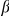-barrel membrane proteins, with only 5 proteins identified at 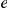-values of 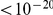. Altogether, only 126 true positives at 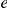-values 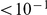 were identified.

**Table 4:** Performance of bbTM matrices in detecting homologs of 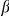-barrel membrane protein sequences from the “true-positive” database.

*e*-value	bbTM_all_	bbTM_in_	bbTM_out_	Blosum62	Phat7573	Slim161	Pam250
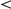 10 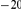	49	62	56	5	48	46	8
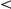 10 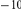	116	106	121	32	121	119	41
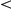 10 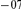	122	121	129	42	133	130	79
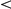 10 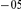	128	127	143	83	141	143	102
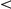 10 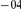	138	131	147	95	148	145	107
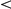 10 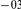	146	139	168	109	176	170	119
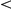 10 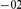	153	144	206	120	200	202	136
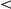 10 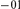	191	166	245	126	272	260	202

Cumulative number of proteins identified as homologs of 20 template 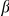-barrel membrane proteins at different 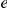-values obtained from Blast searches against the “true-positive” database of 3,079 sequences of 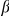-barrel membrane proteins.

Finally, we also performed Blast searches against the non-redundant NCBI database (Table 5). The bbTM matrices retrieved the largest number of hits compared to Phat or the classical Pam matrices. As discussed earlier, the Slim matrices suffer from the problem of very low specificity and would erroneously select many false positives. As expected, the Blosum62 matrix, despite its excellent specificity, performed poorly: only a small number of sequences were identified from the non-redundant NCBI database.

**Table 5:** Performance of bbTM matrices in detecting homologs from the non-redundant NCBI protein sequence database.

*e*-value	bbTM_all_	bbTM_in_	bbTM_out_	Blosum62	Phat7573	Slim161	Pam250
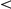 10 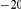	821	934	897	65	605	608	103
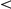 10 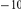	1556	1579	1977	294	1781	1832	416
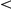 10 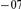	2020	1879	2211	504	2120	2749	649
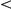 10 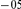	2201	2135	2327	650	2309	4040	812
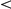 10 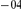	2262	2212	2377	708	2385	5516	1142
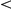 10 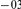	2322	2288	2464	856	2477	7495	1475
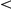 10 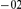	2407	2437	2602	1198	2570	8538	1677
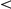 10 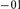	2573	2573	2757	1503	2799	9192	1966

Cumulative number of proteins identified as homologs of the 20 template 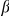-barrel membrane proteins at different 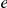-value obtained from Blast searches against the non-redundant NCBI protein database of 13,135,398 sequences.

In summary, the bbTM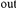 and bbTM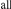 matrices have the best performance among all the matrices tested, with the highest number of “true-positives” detected, while maintaining excellent specificity without erroneously identifying any random sequence, membrane proteins with other architecture, and globular (non-membrane) proteins in our tests at any threshold of 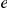-value. Although the classical Blosum62 matrix shows excellent specificity, it has poorer performance in identifying 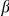-barrel homologous proteins. Among membrane protein specific matrices, Phat retrieves a larger number of true-positive hits, but suffers from the problem of insufficient specificity, as it consistently misidentified random sequences, sequences for other membrane proteins, as well as soluble protein sequences as 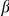-barrel membrane proteins. Slim shows the poorest performance, as it suffers from generating a significant number of false positives.

### Detection of mitochondria membrane proteins

It was estimated that a large number of 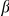-barrel membrane proteins are located at the outer membrane of mitochondria [1], but only four families have been confirmed to date [39]. An interesting question is whether our scoring matrices can be used to detect mitochondria 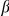-barrel proteins. To answer that question, we performed Blast searches against the non-redundant NCBI database of protein sequences, using transmembrane segments of three different mitochondrial 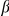-barrel membrane proteins as queries. These are the voltage-dependent anion channel (VDAC), the only mitochondrial porin with known structure; the predicted transmembrane segments of TOM40 [40], the main component of the translocation machinery of mitochondria [41]; and SAM50, an essential component of the sorting and assembly machinery [42]. Using the matrices bbTM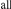, bbTM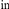, and bbTM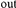, we obtained 266, 277 and 269 homologous proteins, respectively, at the significant level of 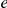-values less than 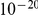, and a total of 383, 379 and 388 at 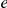-values less than 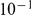. All of these proteins have been verified as mitochondrial proteins by manual inspection of annotations (Table 6).

**Table 6:** Performance of bbTM matrices in detecting homologs of the human mitochondrial proteins VDAC, TOM40 and SAM50.

*e*-value	bbTM_all_	bbTM_in_	bbTM_out_
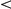 10 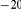	266	277	269
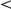 10 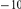	335	324	348
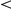 10 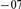	355	354	360
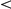 10 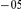	364	361	371
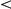 10 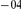	369	364	373
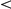 10 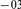	378	370	381
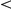 10 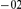	381	376	384
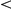 10 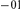	383	379	388

Cumulative number of proteins identified as homologs of the human mitochondrial 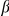-barrel membrane proteins VDAC-1 (uniprot: VDAC1_HUMAN), TOM40 (uniprot: TOM40_HUMAN) and SAM50 (uniprot: SAM50_HUMAN), at different 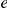-value obtained from Blast searches against the non-redundant NCBI database of 13,135,398 sequences. These hits are all confirmed to be mitochondria proteins by manual inspection of annotation.

### Implications for template-based structure prediction of 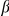-barrel membrane proteins

**Figure 3:**
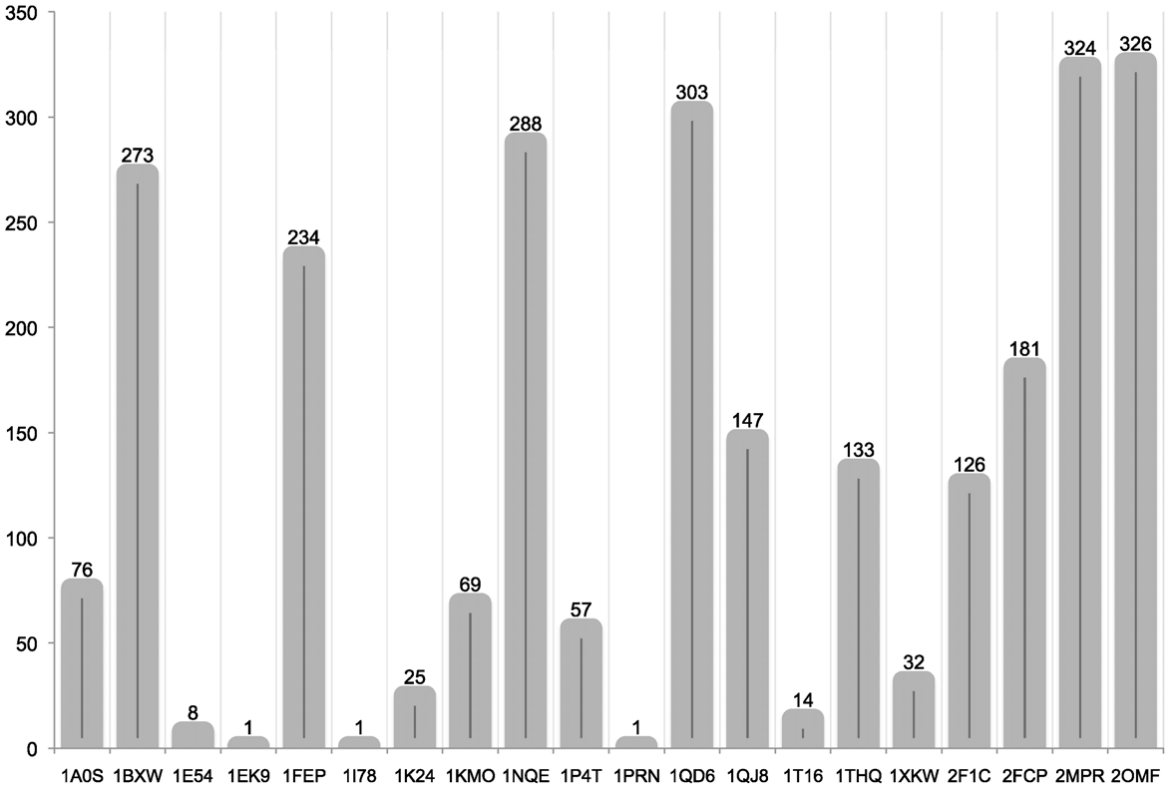
Number of 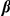-barrel membrane proteins homologous to the 20 proteins with known structures. There are altogether 2,619 proteins in the OMPdb database [44] of 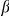-barrel membrane proteins, whose TM regions can be mapped onto one of the 20 proteins by using the bbTM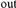 scoring matrix. Structures of the TM regions of these proteins can then be predicted by using template-based structure prediction methods.

An important implication of our results is that we can now reliably detect remote homologs of 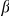-barrel membrane proteins with known structures at genome scale. This will allow prediction of high quality structural models of 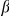-barrel membrane proteins through template-based modeling [43]. Here we estimate the number of 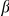-barrel membrane proteins in the OMPdb database [44] whose TM-structures can be modeled reliably through alignments against the template protein structures using the bbTM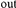 matrix. We found that at the 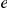-value of less than 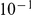 and with at least 75 amino acids to ensure at least 8 transmembrane strands identified, there are a total of 2,619 protein sequences that can be mapped onto one of the 20 known structures we used (Figure 3). On average, each template can be used to model the structures of 131 membrane protein sequences.

## Discussion

### Patterns of amino acid substitutions at lipid interfaces

The estimated substitution rates reveal characteristic patterns common to all 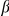-barrel membrane proteins. For residues facing the interior of the barrel, stronger overall sequence conservation is observed. Residues facing the lipid membrane (TM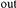) are less conserved and have more substitutions. About twice as many substitutions occur in the TM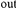 region. However, the pattern of substitution in the TM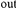 region is very narrow.

The most frequently observed substitutions in this region are among branched aliphatic or small hydrophobic residues (*i.e.*, V-I, V-I, I-L, A-V or A-L), all with very similar physical-chemical properties. Substitutions between aromatic residues (*e.g.*, Y-F and Y-W) are also frequently detected at this interface. Among the aromatics, W has a much larger presence in the TM region (5% in TM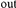, 2% in TM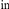) compared to its expected presence in proteins contained in the Uniprot database (1%, data not shown). It is enriched in the aromatic girdles, and has an overall low substitution rate. W likely plays important roles in maintaining the stability or function of 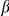-barrel membrane proteins.

Substitutions of polar residues frequently occur among themselves, and also with A, G and V. They are likely to be involved in the maintenance of inter-strand polar-polar motifs as described in a previous study [23]. Some examples of these substitutions can be found in the ferric receptor FepA, the sucrose porin ScrY, the transporters FecA and BtuB, and the ferric hydroxamate uptake receptor FhuA.

With the exception of residue E, ionizable residues in the TM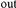 region are mostly found in the lipid-water interface. They are found in large 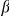-barrel membrane proteins (*e.g.*, ScrY, 18-strands; FepA, 22-strands; BtuB, 22-strands; FhuA, 22-strands; lamB, 18-strands; and OmpF, 16 strands), but not in smaller proteins (*e.g.*, none in OmpA, 8-strands; OmpT, 10-strands; and OmpX with 8-strands).

The overall pattern of substitution of the TM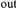 interface suggests that there exists a rich and specific substitution pattern, reflecting strong selection pressure at this interface for amino acids to maintain the same physical-chemical properties. This is perhaps the reason why the bbTM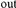 scoring matrices perform the best in identifying remote homologs of 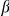-barrel membrane proteins.

### Physical basis of amino acid substitutions in the transmembrane region

There are physical constraints on allowed substitutions due to the requirement of folding and stability of 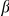-barrel membrane proteins. For example, the membrane environment and the formation of anti-parallel 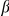-strands are strong constraints that are reflected in the observed substitution pattern.

Anti-parallel strands are arranged with all hydrophobic residues on the side of the barrel facing the lipid interface. Residues L, V, A, F, I and W are frequently found in this interface, which is in agreement with the GES and RW hydrophobicity scales [33], [45], [46]. Under this constraint, these hydrophobic residues are found to mostly exchange among themselves.

The aromatic girdle represents another structural constraint, where W and Y are enriched. Both W and Y residues at the aromatic girdle are important for the 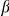-barrel stability, as evidenced by their large TM-region propensities and the frequently occurring spatial motifs of non-H-bonded W-Y interactions [23]. These two residues have very limited substitutions, mostly among themselves. In addition, both aromatic residues may help to facilitate the folding and insertion of the protein into the membrane in a concerted fashion [47], [48].

The result that abundant G is strongly conserved is consistent with the findings from an earlier study, in which it was shown that the substitution of a residue is only weakly influenced by the composition in amino acids, but strongly depends on the constraints of carrying out biological functions and maintaining structural integrity [49]. One example of such constraints is the interaction between G and Y on neighboring strands. In an earlier study, G was found to form strong back-bone H-bonds interactions with aromatic residues. This interstrand interaction, called aromatic rescue [50], likely plays an important role stabilizing these membrane proteins [23].

The lipid-water interface at the end of the 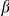-strands also imposes additional constraints, which lead to the placement of many polar residues (S, T, Q or N) and ionizable residues.

Since the interior of the barrel is the location where these proteins interact with ions, metabolites, and substrates, amino acids in this interface are under strong selection pressure to carry out specific biological functions. As a consequence, there are limited substitutions for residues in this interface (Figure 1C).

Aromatic residues facing the TM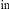 region show a strong conservation as well. Only exchanges between Y-F (21) are observed in this interface, which suggest a strong structural constraint for these residues to be located in specific parts of the interior of the 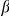-barrel membrane protein, delineating the pathway for substrates across the lumen of the pore or allowing the diffusion of small hydrophobic molecules across the outer membrane [51], [52].

#### Performance Evaluation

Although depicting our results in the form of a Receiver Operating Characteristic (ROC) curve is appealing, there are a number of difficulties that prevent us from using an ROC curve. First, the numbers of true positives and true negatives in any of the data set are not known for each of the query sequences. The total number of sequences in the outer membrane database (3,079) is not the same as the number of true positives when we use only the sequences of a small number of known structures as queries. Second, although the data set of shuffled sequences are most likely to be unrelated to the query proteins, one cannot in principle rule out the presence of some sequences that happens to be homologous to the query sequences by random chance. For these reason, the numbers of true negatives are also not known.

#### Bacterial and mitochondrial 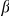-barrel membrane proteins

Despite the relatively remote phylogenetic relationship and overall differences, as the proto-mitochondrion probably entered the primitive eukaryotic cell between two and three billion years ago [7], [53], our results show that matrices derived from bacterial outer membrane proteins can be used to detect mitochondria outer membrane proteins. This is consistent with the observation that 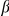-barrel membrane proteins from mitochondria can be readily recognized by the outer membrane insertion machinery of gram negative bacteria [54], and bacterial 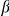-barrel membrane proteins can also be recognized and inserted correctly into the outer membrane of mitochondria [55].

Our finding is consistent with a recent hypothesis that no eukaryote-specific signals for the translocation into mitochondria evolved in mitochondrial 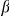-barrel membrane proteins, even though they are now part of eukaryotes. Certain structural elements seems to exist in both mitochondrial and bacterial 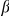-barrel membrane proteins, at least in the TM region, and can be recognized by both insertion machineries [39]. The well-conserved pattern of amino acid substitutions seem to be shared between bacteria and mitochondria membrane proteins, as scoring matrices derived from bacterial membrane proteins are very effective in detecting mitochondrial barrel membrane proteins.

#### Universal substitution patterns

The estimated substitution patterns of residues in the TM region of 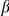-barrel membrane proteins are general. In this study, the 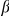-barrel membrane proteins tested in database search for homologs detection are drawn from 19 superfamilies. Despite strong similarity in sequence composition and overall structural similarities, the sequence identity between families is low (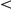
20%). Nevertheless, the scoring matrices can detect remote homologs with excellent specificity and sensitivity. The superfamilies of many of these homologs are not represented by samples from which rates are derived. For example, mitochondria membrane proteins are well detected, which were not used in the estimation of the substitution rates.

Sequences of bacteria and mitochondria are rapidly accumulating from efforts such as metagenomics projects [56]. As the chance of the occurrence of false positives increase significantly when a larger number of bacterial genomic sequences are encountered, avoiding incorrect prediction of 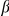-barrel membrane proteins become increasingly important. Existing membrane protein scoring matrices are challenged in this regard. In contrast, the bbTM matrices that we developed are well suited for this task, as they have excellent specificity, with no false positives detected in a large scale database search.

In summary, we have characterized the substitution pattern of residues in the transmembrane segments of 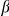-barrel membrane proteins using a continuous time Markov model of amino acid substitution. We found that residues facing both the lipid environment and the interior of the barrel have characteristic patterns. Despite different evolutionary history for different protein families, their substitution patterns are similar. We also derived scoring matrices from estimated substitution rates. In blind tests including both real 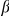-barrel membrane proteins and random sequences of similar composition as control, our scoring matrices can identify remote homologs with excellent specificity and sensitivity. In addition, we have shown that these scoring matrices can be used to detect mitochondrial outer membrane proteins, suggesting that these two classes of membrane proteins share the same pattern of residue substitution throughout evolution. Our results also imply that the structures of the TM segments of a large number of 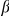-barrel membrane proteins can be predicted reliably based on aligned structural templates.

## Materials and Methods

### Template 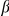-barrel membrane proteins and homologs

We carried out Blast searches [37] using each of the protein sequences of the 20 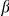-barrel membrane proteins with a solved structure sharing less than 20% pairwise sequence identity as a query against the non-redundant NCBI protein database [57]. For each protein, a multiple sequence alignment was generated using CLUSTALW2 [58]. Regions corresponding to the transmembrane segments were extracted to form the *Transmembrane* 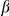 *-Strand Database* (TBSD). Next, using the same query PDB sequences but with only those residues from the transmembrane segments concatenated, we carried out Ssearch searches [59] against the TBSD database. From the output, two sequences for every interval of 10% sequence identity between 90 and 30% were selected, allowing no more than two gaps in every transmembrane segment. This criterion allows us to avoid the problem of over-representations of proteins in a narrow range of evolutionary distance, and enabled selecting sequences exclusively based on the similarity of the transmembrane fragments. This leads to the exclusion of 9 proteins from the set of 20 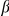-barrel membrane proteins. The final 11 proteins selected are listed in Table 1.

### Estimating amino acid residue substitution rates

#### The Bayesian Monte Carlo method

The substitution rates of residues in the transmembrane segments were estimated following the approach of Tseng and Liang [32]. Briefly, a Bayesian Monte Carlo estimator based on the technique of Markov chain Monte Carlo was used. Estimation is based on the selected set of sequences homologous to the template protein and their phylogenetic trees. The entries 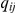 of the substitution rate matrix 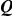 are substitution rates of amino acid residues for the 20 amino acids at an infinitesimally small time interval. Specifically, we have:
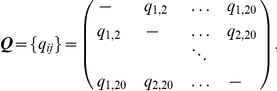
The transition probability matrix of size 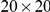 after time 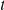 is [60]

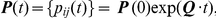
where 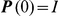. Here 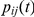 represents the probability that a residue of type 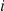 will mutate into a residue of type 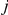 after time 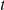.

Using a Bayesian approach, we describe the instantaneous substitution rate 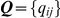 by a posterior distribution 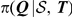, which summarizes the prior information 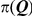 available on the rates 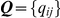 and the likelihood information 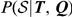 contained in the multiple-alignment 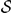 and the phylogenetic tree 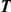. The posterior distribution 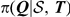 can be estimated using Markov chain Monte Carlo as:
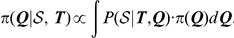
Further details can be found in [32].

In this study, 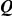 takes the form 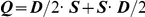, where 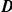 is a diagonal matrix with values taken from the amino acid composition of the set of aligned sequences studied, and 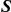 is a symmetric matrix with 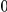 values in diagonal elements, and off-diagonal entries estimated following the model of Adachi et al [61]. Phylogenetic trees 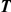 were obtained using the maximum likelihood method Molphy based on the entire length of the protein sequences [61] (see Figure S1 for more details).

#### Valid pairs correction

Once the initial 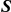 matrix was estimated, we make further corrections to account for different occurring frequency of substitutions appearing in the multiple-aligned sequences [32]. We calculate 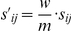, where 
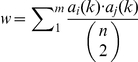
. Here 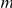 is the total number of columns, 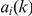 and 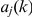 are the number counts of residue 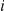 and 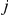 in the 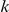-th column of the alignment, respectively, and 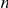 is the number of sequences. We calculated the average 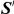 and 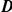 matrices for the 11 proteins used, from which the final rate matrix 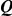 is derived (see Figure S2). This is repeated separately for the aligned sequences of TM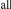, TM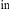, and TM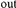.

The 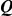 matrix for each of the region is depicted as a bubble plot, in which the area of the circle for the 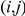-entry is drawn proportional to the value of 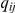 (Figure 1). The scoring matrices at different evolutionary time interval are then derived from the estimated 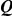 matrix. Further details can be found in references [62], [32]. In this study, we use the scoring matrix of evolutionary time of 40 for bbTM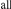 and bbTM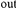, and 36 for bbTM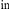, as they give the best discrimination (see Figure S3).

#### Tool availability

We have made available a set of tool to perform Blast searches for 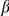-barrel membrane proteins against the non-redundant NCBI database using the bbTM matrices. The URL is at: tanto.bioengr.uic.edu/bbtmst/bbtmstool.php.

## Supporting Information

Figure S1
**Proteins used as structural templates to infer substitution rates.** The 11 proteins and their phylogenetic trees (with labelled homologs) that are used to estimate the substitution rates. We obtain one phylogenetic tree for each of the 11 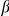-barrel membrane proteins, using the multiple sequence alignment for the entire length of the proteins. The same tree was used for the estimation of three independent substitution rate matrices (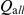, 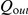, and 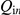).(EPS)

Figure S2
**The estimated instantaneous substitution rates.** Estimating 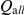. Instantaneous substitution rate values estimated for residues embedded within the outer membrane region (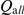). The entries 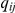 of the rate matrix 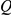 are substitution rates of amino acid residues for the 20 amino acids at an infinitesimally small time interval. The values are in the unit of 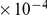 expected residue changes per 100 site between sequences. Estimating 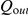. Instantaneous substitution rate values estimated for the subset of residues from the transmembrane segments facing the lipid environment (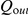). Estimating 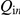. Instantaneous substitution rate values estimated for the subset of residues from the transmembrane segments facing the interior of the barrel (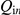).(PDF)

Figure S3
**The bbTM scoring matrices.** Scoring matrix bbTM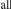. Scoring matrix derived from 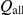 at evolutionary time unit of 40. Scoring matrix bbTM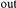. Scoring matrix derived from 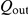 at evolutionary time unit of 40. Scoring matrix bbTM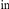. Scoring matrix derived from 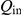 at evolutionary time unit of 36.(PDF)

Dataset S1
**Data set for testing sensitivity and specificity of scoring matrices in detecting homologs of** 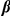 **-barrel membrane proteins:** ***nrbigswiss.fasta*** **.** A set of 2,130 predicted 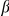-barrel membrane proteins sequences from the PROFtmb database constructed by Bigelow et al [35] with an additional 1,266 sequences annotated as bacterial outer membrane proteins in the Uniprot database [36]. We excluded those proteins with more than 90% identity with the 11 proteins from which we estimated the substitution rates. After removal of redundant sequences, we have a total of 3,079 sequences. This data set is called *dataset_nrbigswiss.fasta*.(FASTA)

Dataset S2
**Data set for testing sensitivity and specificity of scoring matrices in detecting homologs of** 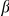 **-barrel membrane proteins:** ***chalmemrandom.fasta*** **.** Random sequences obtained from fully shuffled sequences of 385 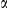-helical and 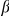-barrel membrane proteins from different organisms. These random sequences preserve the same amino acid composition as membrane proteins. This data set is called *dataset_chalmemrandom.fasta*.(FASTA)

Dataset S3
**Data set for testing sensitivity and specificity of scoring matrices in detecting homologs of** 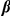 **-barrel membrane proteins:** ***oMBp.fasta*** **.** The *oMBp.fasta* data set (for other MemBrane proteins) were constructed from the Uniprot database [36]. It consists of membrane proteins with a different architecture (non-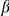-barrel). These were selected based on annotations of “SUBCELLULAR LOCATION: Cell membrane” from *Eukaryota* and *Archaea*. In total, 10,951 protein sequences (1,061 from *Archaea* and 9,890 from *Eukaryota*).(FASTA)

Dataset S4
**Data set for testing sensitivity and specificity of scoring matrices in detecting homologs of** 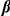 **-barrel membrane proteins:** ***Globular.fasta*** **.** The data set called *Globular.fasta*, consists of 127,485 globular protein sequences from Uniprot with annotations that lack the word “membrane” (16,814 *Archaea* and 110,671 *Eukaryota*). These data sets are available at: tanto.bioengr.uic.edu/bbtmst/.(FASTA)
