# Supplementary material for: Pattern of Amino Acid Substitutions in Transmembrane Domains of β-Barrel Membrane Proteins for Detecting Remote Homologs in Bacteria and Mitochondria
Source: PLoS One. 2011 Nov 1;6(11):e26400. doi: 10.1371/journal.pone.0026400 (PMC3206045; doi:10.1371/journal.pone.0026400)
Supplement: Figure S2 — The estimated instantaneous substitution rates. Estimating . Instantaneous substitution rate values estimated for residues embedded within the outer membrane region (). The entries of the rate matrix are substitution rates of amino acid residues for the 20 amino acids at an infinitesimally small time interval. The values are in the unit of expected residue changes per 100 site between sequences. Estimating . Instantaneous substitution rate values estimated for the subset of residues from the transmembrane segments facing the lipid environment (). Estimating . Instantaneous substitution rate values estimated for the subset of residues from the transmembrane segments facing the interior of the barrel (). (PDF) [file pone.0026400.s002.pdf]

Pattern of Amino Acid Substitutions in Transmembrane Domains of β-Barrel Membrane Proteins for Detecting Remote Homologs in Bacteria and Mitochondria

David Jimenez-Morales[1], Jie Liang[1,*]
1 Department of Bioengineering, University of Illinois at Chicago, Chicago, Illinois, United States of America.
* E-mail: Corresponding author jliang@uic.edu

Figure S2. The estimated instantaneous substitution rates.

A. Estimating Q_{all}. Instantaneous substitution rate values estimated for residues embedded within the outer membrane region (Q_{all}). The entries q_{ij} of the rate matrix Q are substitution rates of amino acid residues for the 20 amino acids at an infinitesimally small time interval. The values are in the unit of $\times 10^{-4}$ expected residue changes per 100 site between sequences.

```
# Q_all: Substitution rate values for the fragments embedded within the outer membrane region.
# Scaling factor: q_ij x 10^-04
```

	S	T	N	Q	D	E	R	K	H	C	P	G	W	Y	F	V	I	L	A	M
S	-91.699	37.585	14.958	2.972	2.530	0.728	1.435	0.998	0.521	0.133	0.197	9.318	0.049	0.240	0.379	0.618	0.162	0.510	17.641	0.723
T	37.585	-94.888	5.927	2.069	0.438	0.741	0.973	1.355	0.246	0.102	0.161	2.930	0.148	0.566	0.385	7.994	1.537	1.461	24.893	5.379
N	14.958	5.927	-37.543	2.511	6.672	0.487	0.695	1.253	0.817	0.037	0.050	1.394	0.102	0.523	0.146	0.162	0.079	0.212	0.958	0.559
Q	2.972	2.069	2.511	-27.419	1.466	5.979	2.735	2.551	3.045	0.133	0.113	0.307	0.113	0.345	0.104	0.576	0.429	0.444	0.741	0.786
D	2.530	0.438	6.672	1.466	-17.105	3.365	0.174	0.350	0.130	0.030	0.042	0.556	0.060	0.123	0.090	0.151	0.071	0.400	0.360	0.096
E	0.728	0.741	0.487	5.979	3.365	-13.688	0.234	0.234	0.052	0.019	0.127	0.443	0.069	0.132	0.049	0.218	0.040	0.142	0.515	0.114
R	1.435	0.973	0.695	2.735	0.174	0.234	-14.180	4.802	1.003	0.061	0.053	0.300	0.071	0.335	0.251	0.223	0.123	0.361	0.262	0.089
K	0.998	1.355	1.253	2.551	0.350	0.234	4.802	-13.533	0.086	0.029	0.042	0.208	0.120	0.123	0.134	0.100	0.142	0.266	0.598	0.143
H	0.521	0.246	0.817	3.045	0.130	0.052	1.003	0.086	-9.029	0.007	0.013	0.124	0.180	2.050	0.112	0.085	0.056	0.295	0.157	0.049
C	0.133	0.102	0.037	0.133	0.030	0.019	0.061	0.029	0.007	-1.104	0.013	0.055	0.016	0.188	0.061	0.071	0.021	0.052	0.046	0.029
P	0.197	0.161	0.050	0.113	0.042	0.127	0.053	0.042	0.013	0.013	-2.418	0.122	0.022	0.053	0.219	0.042	0.027	0.175	0.931	0.016
G	9.318	2.930	1.394	0.307	0.556	0.443	0.300	0.208	0.124	0.055	0.122	-60.010	0.141	0.305	0.595	0.633	0.303	0.855	40.777	0.643
W	0.049	0.148	0.102	0.113	0.060	0.069	0.071	0.120	0.180	0.016	0.022	0.141	-17.997	9.304	3.245	1.482	0.219	2.228	0.304	0.125
Y	0.240	0.566	0.523	0.345	0.123	0.132	0.335	0.123	2.050	0.188	0.053	0.305	9.304	-43.202	24.672	1.237	0.339	1.682	0.646	0.338
F	0.379	0.385	0.146	0.104	0.090	0.049	0.251	0.134	0.112	0.061	0.219	0.595	3.245	24.672	-78.969	7.825	4.858	31.890	2.568	1.387
V	0.618	7.994	0.162	0.576	0.151	0.218	0.223	0.100	0.085	0.071	0.042	0.633	1.482	1.237	7.825	-391.517	194.115	131.115	41.268	3.601
I	0.162	1.537	0.079	0.429	0.071	0.040	0.123	0.142	0.056	0.021	0.027	0.303	0.219	0.339	4.858	194.115	-251.467	44.434	2.718	1.793
L	0.510	1.461	0.212	0.444	0.400	0.142	0.361	0.266	0.295	0.052	0.175	0.855	2.228	1.682	31.890	131.115	44.434	-242.428	9.930	15.977
A	17.641	24.893	0.958	0.741	0.360	0.515	0.262	0.598	0.157	0.046	0.931	40.777	0.304	0.646	2.568	41.268	2.718	9.930	-147.777	2.465
M	0.723	5.379	0.559	0.786	0.096	0.114	0.089	0.143	0.049	0.029	0.016	0.643	0.125	0.338	1.387	3.601	1.793	15.977	2.465	-34.312

B. Estimating Q_{out}. Instantaneous substitution rate values estimated for the subset of residues from the transmembrane segments facing the lipid environment (Q_{out}).

```
# Q_out: Substitution rate values for the subset of residues from the transmembrane segments facing the lipid environment
# Scaling factor: q_ij x 10^-04
```

	S	T	N	Q	D	E	R	K	H	C	P	G	W	Y	F	V	I	L	A	M
S	-21.741	7.444	1.569	0.392	0.295	0.075	0.095	0.141	0.236	0.026	0.314	2.878	0.107	0.485	0.459	0.565	0.226	0.585	5.694	0.155
T	7.444	-56.860	3.098	2.569	0.184	0.462	0.531	0.388	0.134	0.101	0.381	4.389	0.192	1.209	0.348	7.530	0.574	3.180	22.296	1.852
N	1.569	3.098	-11.638	1.381	2.049	0.157	0.276	0.263	0.339	0.043	0.036	0.576	0.062	0.534	0.158	0.198	0.081	0.372	0.181	0.264
Q	0.392	2.569	1.381	-12.734	0.390	0.783	0.825	0.421	2.395	0.070	0.168	0.189	0.205	1.428	0.223	0.415	0.131	0.288	0.253	0.206
D	0.295	0.184	2.049	0.390	-5.754	0.297	0.103	0.273	0.056	0.022	0.030	0.618	0.056	0.366	0.100	0.126	0.075	0.540	0.114	0.058
E	0.075	0.462	0.157	0.783	0.297	-3.888	0.104	0.020	0.042	0.011	0.011	0.227	0.074	0.209	0.139	0.357	0.068	0.346	0.483	0.022
R	0.095	0.531	0.276	0.825	0.103	0.104	-5.535	0.546	0.463	0.019	0.031	0.293	0.115	1.108	0.101	0.191	0.153	0.362	0.173	0.045
D	0.141	0.388	0.263	0.421	0.273	0.020	0.546	-3.532	0.092	0.010	0.056	0.121	0.082	0.290	0.196	0.124	0.074	0.267	0.113	0.055
H	0.236	0.134	0.339	2.395	0.056	0.042	0.463	0.092	-8.410	0.017	0.036	0.088	0.312	3.050	0.158	0.132	0.163	0.465	0.180	0.053
C	0.026	0.101	0.043	0.070	0.022	0.011	0.019	0.010	0.017	-1.396	0.029	0.072	0.046	0.340	0.089	0.231	0.065	0.085	0.104	0.019
P	0.314	0.381	0.036	0.168	0.030	0.011	0.031	0.056	0.036	0.029	-3.905	0.452	0.096	0.077	0.484	0.134	0.083	0.470	0.978	0.037
G	2.878	4.389	0.576	0.189	0.618	0.227	0.293	0.121	0.088	0.072	0.452	-62.493	0.234	0.725	1.682	0.933	0.408	2.405	45.843	0.359
W	0.107	0.192	0.062	0.205	0.056	0.074	0.115	0.082	0.312	0.046	0.096	0.234	-34.252	18.012	5.363	3.532	0.550	4.395	0.595	0.223
Y	0.485	1.209	0.534	1.428	0.366	0.209	1.108	0.290	3.050	0.340	0.077	0.725	18.012	-55.681	19.511	1.881	0.600	3.807	1.434	0.615
F	0.459	0.348	0.158	0.223	0.100	0.139	0.101	0.196	0.158	0.089	0.484	1.682	5.363	19.511	-136.508	21.602	9.725	65.440	7.203	3.528
V	0.565	7.530	0.198	0.415	0.126	0.357	0.191	0.124	0.132	0.231	0.134	0.933	3.532	1.881	21.602	-548.101	275.106	168.339	61.303	5.402
I	0.226	0.574	0.081	0.131	0.075	0.068	0.153	0.074	0.163	0.065	0.083	0.408	0.550	0.600	9.725	275.106	-381.698	81.834	7.808	3.972
L	0.585	3.180	0.372	0.288	0.540	0.346	0.362	0.267	0.465	0.085	0.470	2.405	4.395	3.807	65.440	168.339	81.834	-365.387	16.480	15.727
A	5.694	22.296	0.181	0.253	0.114	0.483	0.173	0.113	0.180	0.104	0.978	45.843	0.595	1.434	7.203	61.303	7.808	16.480	-173.182	1.947
M	0.155	1.852	0.264	0.206	0.058	0.022	0.045	0.055	0.053	0.019	0.037	0.359	0.223	0.615	3.528	5.402	3.972	15.727	1.947	-34.540

C. Estimating Q_{in}. Instantaneous substitution rate values estimated for the subset of residues from the transmembrane segments facing the interior of the barrel (Q_{in}).

Q_in: Substitution rate values for the subset of residues from the transmembrane segments facing the interior of the barrel
Unit: q_ij x 10^-04

	S	T	N	Q	D	E	R	K	H	C	P	G	W	Y	F	V	I	L	A	M
S	-187.806	76.525	25.615	5.620	4.276	2.347	3.360	1.567	1.069	0.270	0.513	19.043	0.493	0.397	0.681	2.342	0.885	0.796	37.999	4.010
T	76.525	-146.250	9.066	4.168	1.006	1.433	1.399	2.166	0.536	0.130	0.232	1.416	0.204	1.582	1.434	12.145	3.521	1.788	22.943	4.557
N	25.615	9.066	-64.924	3.878	9.932	0.719	0.825	2.727	1.206	0.145	0.225	3.216	0.330	0.763	0.216	0.630	0.192	0.901	3.060	1.279
Q	5.620	4.168	3.878	-56.578	3.627	19.786	3.569	5.042	1.264	0.165	0.180	0.538	0.203	0.584	0.282	1.558	1.307	2.204	1.796	0.809
D	4.276	1.006	9.932	3.627	-41.038	14.389	0.352	0.291	0.207	0.048	0.136	2.966	0.159	0.248	0.383	0.482	0.134	0.893	1.136	0.372
E	2.347	1.433	0.719	19.786	14.389	-47.877	0.356	0.428	0.168	0.220	0.278	1.002	0.443	0.311	0.203	1.886	0.184	0.440	2.496	0.788
R	3.360	1.399	0.825	3.569	0.352	0.356	-23.582	7.702	1.449	0.174	0.076	0.876	0.085	0.302	0.587	0.717	0.220	0.692	0.664	0.178
D	1.567	2.166	2.727	5.042	0.291	0.428	7.702	-23.477	0.272	0.050	0.139	0.398	0.162	0.101	0.117	0.226	0.307	0.561	1.084	0.138
H	1.069	0.536	1.206	1.264	0.207	0.168	1.449	0.272	-7.364	0.012	0.018	0.242	0.040	0.344	0.060	0.075	0.045	0.193	0.134	0.031
C	0.270	0.130	0.145	0.165	0.048	0.220	0.174	0.050	0.012	-1.861	0.007	0.150	0.016	0.181	0.029	0.067	0.019	0.038	0.118	0.020
P	0.513	0.232	0.225	0.180	0.136	0.278	0.076	0.139	0.018	0.007	-3.094	0.234	0.066	0.116	0.053	0.065	0.038	0.087	0.593	0.039
G	19.043	1.416	3.216	0.538	2.966	1.002	0.876	0.398	0.242	0.150	0.234	-83.068	0.331	0.302	0.268	0.616	0.337	0.561	50.062	0.509
W	0.493	0.204	0.330	0.203	0.159	0.443	0.085	0.162	0.040	0.016	0.066	0.331	-3.887	0.737	0.111	0.063	0.052	0.132	0.188	0.071
Y	0.397	1.582	0.763	0.584	0.248	0.311	0.302	0.101	0.344	0.181	0.116	0.302	0.737	-28.516	21.291	0.310	0.246	0.265	0.259	0.178
F	0.681	1.434	0.216	0.282	0.383	0.203	0.587	0.117	0.060	0.029	0.053	0.268	0.111	21.291	-29.099	0.302	0.356	1.117	0.427	1.184
V	2.342	12.145	0.630	1.558	0.482	1.886	0.717	0.226	0.075	0.067	0.065	0.616	0.063	0.310	0.302	-53.401	15.204	7.038	8.938	0.737
I	0.885	3.521	0.192	1.307	0.134	0.184	0.220	0.307	0.045	0.019	0.038	0.337	0.052	0.246	0.356	15.204	-28.079	2.607	1.458	0.967
L	0.796	1.788	0.901	2.204	0.893	0.440	0.692	0.561	0.193	0.038	0.087	0.561	0.132	0.265	1.117	7.038	2.607	-32.970	7.617	5.037
A	37.999	22.943	3.060	1.796	1.136	2.496	0.664	1.084	0.134	0.118	0.593	50.062	0.188	0.259	0.427	8.938	1.458	7.617	-143.667	2.696
M	4.010	4.557	1.279	0.809	0.372	0.788	0.178	0.138	0.031	0.020	0.039	0.509	0.071	0.178	1.184	0.737	0.967	5.037	2.696	-23.602
