# Supplementary material for: Pattern of Amino Acid Substitutions in Transmembrane Domains of β-Barrel Membrane Proteins for Detecting Remote Homologs in Bacteria and Mitochondria
Source: PLoS One. 2011 Nov 1;6(11):e26400. doi: 10.1371/journal.pone.0026400 (PMC3206045; doi:10.1371/journal.pone.0026400)
Supplement: Figure S3 — The bbTM scoring matrices. Scoring matrix bbTM. Scoring matrix derived from at evolutionary time unit of 40. Scoring matrix bbTM. Scoring matrix derived from at evolutionary time unit of 40. Scoring matrix bbTM. Scoring matrix derived from at evolutionary time unit of 36. (PDF) [file pone.0026400.s003.pdf]

Pattern of Amino Acid Substitutions in Transmembrane Domains of β-Barrel Membrane Proteins for Detecting Remote Homologs in Bacteria and Mitochondria

David Jimenez-Morales[1], Jie Liang[1,*]
1 Department of Bioengineering, University of Illinois at Chicago, Chicago, Illinois, United States of America.
* E-mail: Corresponding author jliang@uic.edu

Figure S3. The bbTM scoring matrices.

A. Scoring matrix bbTM$_{\text{all}}$. Scoring matrix at evolutionary time unit of 40 derived from Q_{all}. Note: The last line denotes the lowest score in all columns and rows to conform with format of Blosum and Pam for use in programs such as ClustalW.

```
# bbTMall
# Lowest score = -24 , Highest score = 24
    A   R   N   D   C   Q   E   G   H   I   L   K   M   F   P   S   T   W   Y   V   B   Z   X   *
A   6 -11  -7 -10 -19  -9 -10   1 -12  -2  -2  -9  -5  -5  -8  -1   0 -10 -10   0  -8  -4  -6 -24
R -11  10  -7 -11 -13  -4 -10 -12  -5 -12 -11  -1 -12 -11 -14  -6  -8 -14 -11 -12  -9 -11  -8 -24
N  -7  -7  10   0 -13  -4  -8  -7  -6 -12 -12  -5  -7 -12 -14   0  -2 -13 -10 -11   5  -7  -7 -24
D -10 -11   0  11 -13  -5  -2 -10 -10 -13 -11  -9 -12 -14 -14  -4  -8 -14 -14 -12   6  -6  -8 -24
C -16 -13 -14 -14  24 -11 -16 -16 -16 -15 -16 -14 -13 -14 -14 -12 -13 -16 -12 -15 -14 -16 -12 -24
Q  -9  -4  -4  -5 -11  10  -1 -11  -2  -9 -10  -4  -7 -13 -12  -4  -5 -13 -11  -9  -4  -6  -6 -24
E -10 -10  -8  -2 -24  -1  10 -11 -12 -14 -13 -10 -12 -15 -11  -8  -8 -14 -14 -12  -5  -0  -9 -24
G   1 -12  -7 -10 -24 -11 -11   7 -14  -8  -8 -12  -8 -10 -13  -2  -4 -13 -12  -6  -8  -2  -8 -24
H -12  -5  -6 -10 -24  -2 -12 -14  13 -13 -12 -11 -12 -11 -16  -9 -10 -10  -5 -13  -8 -13  -9 -24
I  -2 -12 -12 -13 -17  -9 -14  -8 -13   7   3 -12  -3  -2 -14  -9  -5  -8  -9   5 -12 -11  -7 -24
L  -2 -11 -12 -11 -18 -10 -13  -8 -12   3   6 -12   0   1 -13  -9  -6  -6  -6   3 -11 -10  -6 -24
K  -9  -1  -5  -9 -13  -4 -10 -12 -11 -12 -12  11 -11 -13 -14  -7  -6 -12 -14 -12  -7 -11  -8 -24
M  -5 -12  -7 -12 -13  -7 -12  -8 -12  -3   0 -11  12  -5 -15  -7  -2 -10 -10  -2  -9 -10  -7 -24
F  -5 -11 -12 -14 -13 -13 -15 -10 -11  -2   1 -13  -5   8 -11 -10  -9  -3   1  -1 -13 -12  -7 -24
P  -8 -14 -14 -14 -13 -12 -11 -13 -16 -15 -13 -14 -15 -11  14 -11 -12 -16 -16 -13 -14 -12 -11 -24
S  -1  -6   0  -4 -13  -4  -8  -2  -9  -9  -9  -7  -7 -10 -11   8   2 -15 -12  -7  -2  -5  -6 -24
T   0  -8  -2  -8 -13  -5  -8  -4 -10  -5  -6  -6  -2  -9 -12   2   8 -12 -10  -3  -5  -6  -5 -24
W -10 -14 -13 -14 -22 -13 -14 -13 -10  -8  -6 -12 -10  -3 -16 -15 -12  11  -1  -6 -13 -13 -10 -24
Y -10 -11 -10 -14 -13 -11 -14 -12  -5  -9  -6 -14 -10   1 -15 -12 -10  -1   7  -8 -12 -13  -8 -24
V   0 -12 -11 -12 -14  -9 -12  -6 -13   5   3 -12  -2  -1 -13  -7  -3  -6  -8   5 -11  -9  -6 -24
B  -8  -9   5   6 -14  -4  -5  -8  -8 -12 -11  -7  -9 -13 -14  -2  -5 -13 -12 -11  11  -4  -7 -24
Z  -4 -11  -7  -6 -16  -6  -0  -2 -13 -11 -10 -11 -10 -12 -12  -5  -6 -13 -13  -9  -4  10  -7 -24
X  -6  -8  -7  -8 -12  -6  -9  -8  -9  -7  -6  -8  -7  -7 -11  -6  -5 -10  -8  -6  -7  -7   1 -24
* -24 -24 -24 -24 -24 -24 -24 -24 -24 -24 -24 -24 -24 -24 -24 -24 -24 -24 -24 -24 -24 -24 -24   1
```

B. Scoring matrix bbTM$_{out}$. Scoring matrix at evolutionary time unit of 40 derived from Q_{out}.

```
# bbTMout
# Lowest score = -16 , Highest score = 25
    A   R   N   D   C   Q   E   G   H   I   L   K   M   F   P   S   T   W   Y   V   B   Z   X   *
A   6 -12 -11 -13 -12 -11 -10   1 -13  -1  -1 -13  -5  -3  -8  -3   0  -9  -8   0 -12  -4  -6 -16
R -12  14  -7  -9 -12  -5  -8 -10  -6 -12 -12  -4 -13 -13 -14 -11  -8 -12  -8 -12  -8  -9  -8 -16
N -11  -7  13  -1 -12  -3  -8  -9  -7 -13 -12  -7  -8 -12 -14  -3  -3 -14 -10 -12   6  -8  -7 -16
D -13  -9  -1  14 -12  -7  -5  -8 -12 -13 -11  -6 -12 -13 -14  -8 -11 -14 -11 -13   7  -6  -8 -16
C -14 -13 -11 -12  25 -11 -11 -14 -14 -14 -15 -14 -14 -14 -13 -14 -12 -14 -11 -13 -11 -12 -11 -16
Q -11  -5  -3  -7 -10  12  -4 -12  -2 -12 -12  -6  -9 -12 -10  -8  -4 -10  -8 -11  -5  -8  -7 -16
E -10  -8  -8  -5 -12  -4  17 -11 -12 -12 -12 -13 -14 -12 -16 -11  -8 -13 -13 -11  -6   3  -9 -16
G   1 -10  -9  -8 -12 -12 -11   8 -14  -6  -6 -13  -9  -7  -9  -4  -3 -11 -10  -5  -8  -1  -7 -16
H -13  -6  -7 -12 -12  -2 -12 -14  13 -12 -12 -10 -13 -11 -14  -9 -12  -9  -5 -13  -9 -13  -9 -16
I  -1 -12 -13 -13 -13 -12 -12  -6 -12   5   2 -14  -3  -1 -13  -9  -5  -6  -8   3 -13  -9  -7 -16
L  -1 -12 -12 -11 -15 -12 -12  -6 -12   2   4 -13  -2   1 -11  -9  -5  -5  -6   2 -11  -9  -6 -16
K -13  -4  -7  -6 -12  -6 -13 -13 -10 -14 -13  14 -12 -12 -12 -10  -9 -13 -12 -13  -6 -13  -9 -16
M  -5 -13  -8 -12 -12  -9 -14  -9 -13  -3  -2 -12  12  -3 -13 -10  -4  -9  -9  -3 -10 -11  -7 -16
F  -3 -13 -12 -13 -12 -12 -12  -7 -11  -1   1 -12  -3   6 -10 -10  -8  -3  -2   0 -12  -9  -6 -16
P  -8 -14 -14 -14 -12 -10 -16  -9 -14 -13 -11 -12 -14 -10  12  -8  -9 -13 -15 -12 -14 -12 -10 -16
S  -3 -11  -3  -8 -12  -8 -11  -4  -9  -9  -9 -10 -10 -10  -8  11  -1 -12 -11  -8  -5  -7  -7 -16
T   0  -8  -3 -11 -12  -4  -8  -3 -12  -5  -5  -9  -4  -8  -9  -1   9 -11  -8  -3  -7  -5  -5 -16
W  -9 -12 -14 -14 -12 -10 -13 -11  -9  -6  -5 -13  -9  -3 -13 -12 -11   9  -1  -5 -14 -12  -8 -16
Y  -8  -8 -10 -11 -12  -8 -13 -10  -5  -8  -6 -12  -9  -2 -15 -11  -8  -1   6  -7 -10 -11  -7 -16
V   0 -12 -12 -13 -13 -11 -11  -5 -13   3   2 -13  -3   0 -12  -8  -3  -5  -7   4 -12  -8  -6 -16
B -12  -8   6   7 -11  -5  -6  -8  -9 -13 -11  -6 -10 -12 -14  -5  -7 -14 -10 -12  14  -5  -8 -16
Z  -4  -9  -8  -6 -12  -8   3  -1 -13  -9  -9 -13 -11  -9 -12  -7  -5 -12 -11  -8  -5  15  -8 -16
X  -6  -8  -7  -8 -11  -7  -9  -7  -9  -7  -6  -9  -7  -6 -10  -7  -5  -8  -7  -6  -8  -8   1 -16
* -16 -16 -16 -16 -16 -16 -16 -16 -16 -16 -16 -16 -16 -16 -16 -16 -16 -16 -16 -16 -16 -16 -16   1
```

C. Scoring matrix bbTM$_{in}$. Scoring matrix at evolutionary time unit of 36 derived from Q_{in}.

```
#bbTMin
# Lowest score = -14 , Highest score = 23
    A   R   N   D   C   Q   E   G   H   I   L   K   M   F   P   S   T   W   Y   V   B   Z   X   *
A   7  -9  -4  -7 -14  -7  -6   1 -12  -6  -3  -8  -5 -10  -9   0   0 -12 -12  -2  -5  -2  -5 -14
R  -9   8  -8 -11 -12  -5 -11 -10  -6 -11  -9  -2 -12  -9 -14  -6  -7 -14 -11  -8  -9 -10  -8 -14
N  -4  -8   8  -1 -12  -4  -8  -6  -6 -10  -8  -5  -6 -11 -11   0  -2 -10  -9  -8   4  -7  -6 -14
D  -7 -11  -1   9 -14  -4   0  -6 -10 -12  -7 -10  -9 -10 -12  -5  -7 -11 -11  -8   4  -3  -7 -14
C -13 -12 -12 -14  23 -12 -11 -14 -14 -14 -14 -14 -14 -14 -14 -12 -13 -14 -11 -12 -13 -12 -11 -14
Q  -7  -5  -4  -4 -12   8   0 -10  -6  -6  -5  -3  -8 -11 -12  -4  -5 -11 -10  -6  -4  -5  -6 -14
E  -6 -11  -8   0 -11   0   8  -9 -12 -11 -10 -10  -8 -12 -11  -7  -7 -10 -11  -6  -4  -0  -7 -14
G   1 -10  -6  -6 -14 -10  -9   6 -13 -11  -9 -11  -9 -13 -13  -2  -5 -12 -13  -8  -6  -1  -8 -14
H -12  -6  -6 -10 -14  -6 -12 -13  14 -13 -10 -10 -14 -13 -14  -8  -9 -13  -9 -12  -8 -12  -9 -14
I  -6 -11 -10 -12 -14  -6 -11 -11 -13  12  -3 -10  -5  -8 -13  -8  -4 -13 -10   3 -11 -11  -7 -14
L  -3  -9  -8  -7 -14  -5 -10  -9 -10  -3  10  -9  -1  -6 -12  -8  -6 -12 -11  -1  -7  -9  -6 -14
K  -8  -2  -5 -10 -14  -3 -10 -11 -10 -10  -9   9 -12 -13 -12  -7  -6 -12 -14 -10  -7 -10  -8 -14
M  -5 -12  -6  -9 -14  -8  -8  -9 -14  -5  -1 -12  12  -5 -13  -4  -3 -12 -11  -6  -7  -8  -7 -14
F -10  -9 -11 -10 -14 -11 -12 -13 -13  -8  -6 -13  -5  11 -13  -9  -7 -11   2  -9 -10 -12  -8 -14
P  -9 -14 -11 -12 -14 -12 -11 -12 -14 -13 -12 -12 -13 -13  16 -10 -11 -11 -12 -12 -11 -11 -10 -14
S   0  -6   0  -5 -12  -4  -7  -2  -8  -8  -8  -7  -4  -9 -10   6   2 -10 -11  -5  -2  -4  -5 -14
T   0  -7  -2  -7 -14  -5  -7  -5  -9  -4  -6  -6  -3  -7 -11   2   7 -12  -7  -1  -4  -6  -5 -14
W -12 -14 -10 -11 -14 -11 -10 -12 -13 -13 -12 -12 -12 -11 -11 -10 -12  13  -7 -13 -10 -11 -10 -14
Y -12 -11  -9 -11 -11 -10 -11 -13  -9 -10 -11 -14 -11   2 -12 -11  -7  -7   9 -10 -10 -12  -8 -14
V  -2  -8  -8  -8 -12  -6  -6  -8 -12   3  -1 -10  -6  -9 -12  -5  -1 -13 -10  11  -8  -7  -6 -14
B  -5  -9   4   4 -13  -4  -4  -6  -8 -11  -7  -7  -7 -10 -11  -2  -4 -10 -10  -8   9  -4  -6 -14
Z  -2 -10  -7  -3 -12  -5  -0  -1 -12 -11  -9 -10  -8 -12 -11  -4  -6 -11 -12  -7  -4   8  -6 -14
X  -5  -8  -6  -7 -11  -6  -7  -8  -9  -7  -6  -8  -7  -8 -10  -5  -5 -10  -8  -6  -6  -6   1 -14
* -14 -14 -14 -14 -14 -14 -14 -14 -14 -14 -14 -14 -14 -14 -14 -14 -14 -14 -14 -14 -14 -14 -14   1
```
